# Comparison of echocardiography and Cine Magnetic Resonance Imaging for the measurement of left ventricular wall thickness and ventricular dimension - Are they interchangeable?

**DOI:** 10.1186/1532-429X-16-S1-P283

**Published:** 2014-01-16

**Authors:** Takashi Tanimoto, Shingo Ota, Yamano Takashi, Yoshiki Matsuo, Yasushi Ino, Tomoyuki Yamaguchi, Kumiko Hirata, Takashi Kubo, Toshio Imanishi, Takashi Akasaka

**Affiliations:** 1 Cardiovascular Medicine, Wakayama Mecical University, Wakayama, Japan

## Background

In the assessment of left ventricular morphology and function, measurements of left ventricular wall thickness and dimension are essential in daily practice. Although the normal values of left ventricular wall thickness and dimension by 2-dimensional echocardiography had been reported, normal values for cine MRI measurements and the relationship with echocardiography are still unknown. This study investigated whether the results of each technique are interchangeable.

## Methods

A total of 55 normal subjects (24 male and 31 female; age range, 19-78) underwent both 2-dimensional echocardiography and cine MRI. Left ventricular wall thickness of interventricular septum (IVS) and posterior wall (PW), diastolic dimension (Dd), and systolic dimension (Ds) were measured at end-diastole in parasternal long axis view by echocardiography. For cine MRI examination, all parameters were measured in the 3-chamber view of the left ventricle at the mitral leaflet tips, as recommended by the society of cardiovascular magnetic resonance guideline.

## Results

For wall thickness analysis, the respective mean values obtained by echocardiography and cine MRI were as follows: IVS, 8.7 ± 1.5 mm and 8.0 ± 3.0 mm (p = 0.12); PW, 8.6 ± 1.4 mm and 4.6 ± 1.7 mm (p < 0.001), IVS/PW ratio, 1.0 ± 0.1 and 1.8 ± 0.6 (p < 0.001). For left ventricular dimension analysis, the mean echocardiographic and MRI values were as follows: Dd, 44.4 ± 5.1 mm and 49.1 ± 4.9 mm (p < 0.001); Ds, 29.5 ± 4.7 mm and 32.4 ± 4.6 mm (p = 0.001). Finally, mean values of IVS+Dd+PW were 61.6 ± 6.5 mm by echocardiography and 61.9 ± 7.4 mm by MRI (p = 0.82), which indicates that the identification of endocardial border of posterior wall are different between echocardiography and cine MRI.

## Conclusions

Normal values of cine MRI measurements of left ventricle in healthy population are reported. These results suggest that PW thickness and ventricular dimensions are not interchangeable between echocardiography and MRI.

## Funding

I have nothing to disclose.

